# Detection of human herpes viruses 1-5 in miscarriage: A case-control study

**DOI:** 10.18502/ijrm.v13i7.7367

**Published:** 2020-07-22

**Authors:** Javad Charostad, Talat Mokhtari-Azad1 D.V.M, MPH, Ph.D., Jila Yavarian, Nastaran Ghavami, Seyed Mahmood Seyed Khorrami, Emad Behboudi, Somayeh Jalilvand, Somayeh Shatizadeh Malekshahi, Nazanin Zahra Shafiei-Jandaghi

**Affiliations:** ^1^Virology Department, School of Public Health, Tehran University of Medical Sciences, Tehran, Iran.; ^2^Department of Virology, Faculty of Medical Sciences, Tarbiat Modares University, Tehran, Iran.

**Keywords:** Spontaneous abortion, Therapeutic abortion, Infections, Human herpes viruses.

## Abstract

**Background:**

Miscarriage is the spontaneous pregnancy loss before 24 wk of gestation. The incidence rate of miscarriage over the past few decades has shown steady or even growing trends. Viral intrauterine infections are one of the probable etiological causes of miscarriage. Previous evidence have shown that human herpes viruses (HHVs) could be considered as the potential reasons for intrauterine infections and adverse pregnancy outcomes.

**Objective:**

This case-control study aimed to detect HHV1-5 DNAs in placental tissues and assess their association with miscarriage during the first 24 wk of pregnancy in spontaneous and therapeutic abortions.

**Materials and Methods:**

Placental tissues from 83 women with spontaneous abortions during the first and the second trimesters of pregnancy and 81 women with therapeutic abortion during the same gestational age were collected. The DNA extraction was performed by the phenol/chloroform method. A part of the DNA polymerase gene of HHVs was amplified with multiplex nested-polymerase chain reaction. The polymerase chain reaction products were subjected to sequencing.

**Results:**

The results showed the presence of human cytomegalovirus genome in the placenta of both spontaneous (8.4%) and therapeutic (4.9%) abortions. No statistically significant differences were found between these two groups. The other investigated viruses were not detected here.

**Conclusion:**

In conclusion, like some other studies, no correlation was detected between the HHVs placental infections and the increased risk of spontaneous abortions. In order to find the actual role of HHVs infections in miscarriage, further investigations should be performed on a larger sample size in different areas.

## 1. Introduction

Miscarriage is defined as a spontaneous abortion within the first 24 wk of gestation. It is one of the most frequent adverse pregnancy outcomes, the incidence of which has shown steady or even growing trends over the past few decades despite the extensive efforts in prenatal care (1-3). Approximately, 12-15% of clinically established pregnancies lead to miscarriage (3). Viral intrauterine infections are among the key etiological causes of spontaneous abortions (4). Previous studies have shown that human herpes viruses (HHVs) including HHV1/2 (herpes simplex viruses; HSV1/2), HHV3 (varicella zoster virus; VZV), HHV4 (Epstein-Barr virus; EBV), and HHV5 (human cytomegalovirus; HCMV) regularly cause intrauterine infections and lead to adverse pregnancy outcomes (4-7). Among HHVs, HCMV has been studied extensively, due to its highest prevalence in intrauterine infections (8). The placenta is a critical barrier between fetal and maternal blood circulation. The integrity of this barrier could be affected by transplacental transmission of some viruses such as HCMV (9, 10). It appears that HCMV infection may impair the differentiation of cytotrophoblasts (11, 12), which may lead to miscarriages and fetus abnormalities (10, 13).

Despite these evidence, the association of HCMV placental infection with miscarriage has not been fully clarified (14). In addition to HCMV, intrauterine infections by HSV1/2can lead to adverse consequences including miscarriage (15). Another member of HHVs, EBV, tropism to trophoblasts in the placenta (15, 16); however, the relationship between EBV infection and abortion remains controversial (17). Meanwhile, VZV can spread to the fetus via placental intrauterine infection, so the increased risk of miscarriage due to this infection has been investigated by some researchers (18). To date, several studies have been conducted to determine the effects of HSV1/2, VZV, EBV, and HCMV on pregnancy outcomes separately. But only a few studies have examined all of them simultaneously.

As mentioned before, viral infections are among the leading causes of spontaneous abortions, this case-control study concentrates on the detection of HHV1-5 DNAs in placental tissues and finding their association with miscarriage during the first 24 wk of pregnancy by evaluating spontaneous abortions as the case group and therapeutic abortions as the control group.

## 2. Materials and Methods

### Patients, specimens and DNA extraction

In this case-control study, a total of 164 fresh frozen placental tissues from abortion cases of 4-24 wk of pregnancy were collected from two hospitals in Tehran (Mother and Fetus Research Center of Emam Khomeini Hospital and Arash Hospital) during April 2014 to April 2015. Of the 164 cases, 83 were women with spontaneous abortions (case group) aged 18-45 yr and 81 cases were women with therapeutic abortions (control group) aged 19-41 yr. As intentional abortion (without any therapeutic reason) is illegal in Iran, the placental tissues of women who underwent legal pregnancy termination were collected for the control group. The inclusion criteria for this group were pregnancy termination due to medical indications of legal therapeutic abortions which could not be related to HHVs infections as follows: (1) where the life of the mother was in danger because of the pregnancy and (2) in cases of anomalies in the fetus that made it not viable (e.g., anencephaly) or caused troubles for a mother to look after it after birth (e.g., major thalassemia). Therapeutic abortions which could be related to HHVs infections were excluded from the control group in this study.

For the case group, specimens were obtained from the placentas of women with spontaneous pregnancy loss. The most consistent clinical symptoms of case group were abdominal pain and bleeding from the uterus. The DNA was extracted from 0.5gr of each placental sample by proteinase K digestion and the phenol/chloroform method. Briefly, placental samples were deparaffinized with xylene and resuspended in lysis buffer and treated overnight with proteinase K (20mg/ml). Then, genomic DNA was extracted by phenol/chloroform method followed by ethanol precipitation and dissolved in TE buffer. All extractions were aliquoted and preserved at -70°C.

### Molecular assays

The quality of each extraction was tested by polymerase chain reaction (PCR) for human β globin gene using specific primers as described previously (19). To detect the genomes of HHVs 1-5, multiplex nested PCRs (20) were performed on all positive samples in β globin PCR. The clinical samples with a positive result for HSV1/2, VZV, EBV, and HCMV from former studies were used for positive controls, and double distilled water was used as the negative control. The PCR products of positive cases in the second rounds of nested-PCR were subjected to sequencing. To confirm the species of identified HHVs, sequences were analyzed with BioEdit version 7.0.0 DNA analysis software (19) and nucleotide BLAST.

### Ethical consideration

This study was approved by the ethics committee (IR.TUMS.VCR.REC.1395.42). No patient identifying information was included in this manuscript. Data related to mother's age, the number of pregnancies and abortions and gestational age were collected through a questionnaire filled at the time of sample collection. The participants were informed about the study goals and oral consent was taken from each of them.

### Statistical analysis

Data analysis was performed using the statistical package of SPSS for Windows version 16 (SPSS Inc., Chicago IL). To assess the difference between case and control groups, Chi-square test, Fisher exact test and Independent *t* test were used. P <0.05 was considered statistically significant.

## 3. Results

### Demographic characteristics 

Placental tissues from 83 women who experienced spontaneous abortions (case group) and 81 women who experienced therapeutic abortions (control group) were investigated for the presence of HSV-1/2, VZV, EBV, and HCMV. Similar features were observed both in the case and control groups regarding maternal age and the number of abortions. The mean maternal age in both groups was around 29 years (SD= 5).The number of abortions in case and control groups was not significantly different (p = 0.49).

### Viral DNA detection

Of the 164 placental tissues studied in this case-control study, HCMV DNA was detected only in 11 cases (6.7%), of which 7 cases (8.4%) were in the spontaneous abortion group and 4 cases (4.9%) in the therapeutic abortion group (p = 0.535). The other investigated HHVs were not detected here. The information about all variables related to HCMV-positive samples are mentioned in Table I. No statistically significant correlation between HCMV positivity and maternal age (p = 0.34), gestational age (p = 0.27), number of abortions (p = 1.00), and the type of abortion (therapeutic vs spontaneous abortion) (p = 0.53) was found. Moreover, there were 12 recurrent miscarriage cases and none of them were positive for HHV 1-5 DNAs.

**Table 1:** Variables in HCMV-positive samples


	**Variables**			
**HCMV-positive samples**	**Maternal age**	**Gestational age (wk)**	Type of abortion*	**Number of previous abortions**
	**1**	20	6	SA	0
**2**	34	6	SA	0
**3**	27	6	TA	0
**4**	28	6	TA	0
**5**	22	8	SA	0
**6**	33	9	SA	0
**7**	21	10	SA	0
**8**	32	12	SA	1
**9**	35	14	TA	2
**10**	29	18	TA	1
**11**	27	19	SA	0
HCMV: Human cytomegalovirus; SA: Spontaneous abortion; TA: Therapeutic abortion				

## 4. Discussion

The present study evaluated and compared the prevalence of HHV1-5 DNAs in placental tissues of pregnant women with spontaneous vs. therapeutic abortions to assess their probable role in spontaneous abortion induction. Among these viruses, only the genome of HCMV was detected in the placenta of spontaneous (8.4%) and therapeutic (4.9%) abortions. Although the frequency of HCMV DNA detection was higher in the spontaneous abortions compared to the therapeutic ones, no significant association was found between the HCMV DNA detection in the placenta and the increased risk of miscarriage. Different studies have shown that certain intrauterine infections were recognized or assumed to play a role in miscarriage, the exact mechanism of which is still not fully clarified (4). Similar to the current study, a number of studies have attempted to reveal a possible correlation between miscarriage and HHVs placental infections in pregnant women. Some of these reports concluded similarly and some showed different results compared to the present study. Similar to this study, other studies have reported a higher rate of HCMV detection compared to the other HHVs in the placenta.

Al-Buhtori and colleagues evaluated 28 placental tissues from spontaneous abortion cases; they detected HCMV in 10 cases (35.7%) while 3 of them were co-infected with HSV1/2. No genome of VZV and EBV were found in their study (21). In a study conducted in China, HCMV DNA in cervicovaginal secretions was evaluated in 624 normal pregnancies and 440 miscarriages. HCMV DNA was detected in 10.9% and 8.5% of cases who had miscarriages and normal pregnancies, respectively. These findings suggested that HCMV infection of the genital tract in first trimester of pregnancy may affect pregnancy outcome and cause miscarriage (14).

Moreover, these findings were supported in a review article published in 2016 that named HCMV among the infectious agents which could increase the risk of miscarriage (4). However, similar to our findings, some other studies could not show any association between HCMV placental infection and increased risk of miscarriages. Gao and colleagues in China investigated HCMV spontaneous abortion cases in the first 12 wk of pregnancy. In their study, no HCMV DNA was detected in the placenta samples (22). In 2018, another Chinese research group investigated HCMV placental infection in miscarriages that occurred during the first trimester of pregnancy. They also could not find any significant correlation between infection and miscarriage as 9.1% of miscarriage cases (3/33) and 6.7% of normal cases (2/30) were positive for HCMV (14).

Formerly it was shown that the role of intrauterine infections in recurrent abortions was unclear, with a probable incidence of 0.5-5% (4). In a case-control study of women with recurrent pregnancy loss, anti-HCMV IgG and IgM and IgG avidity index were evaluated. They found that previous exposure to HCMV was significantly higher in patients with recurrent pregnancy loss than the control groups. However, no association was found between the IgG avidity index and recurrent pregnancy loss (23). In the present investigation, of the 12 recurrent miscarriage cases none were positive for HHV 1-5 DNAs. It should be noted that the rate of HCMV abortions was unclear (IgG positivity) in Iran and is estimated at around 90-98%in adults (24-26). So, the rate of HCMV primary infection during pregnancy would be low. However, the immunosuppression state during this period could result in HCMV reactivation, intrauterine fetal infection, and abnormalities in the fetus (8, 27).

As stated, in the present research, no HSV1/2, VZV, and EBV genomes were detected in 164 placental tissues. Previously, it has been shown that in spite of EBV reactivation during pregnancy, the rate of intrauterine infection remained low and miscarriage due to EBV infection was not reported. Haeri and colleagues examined EBV reactivation in serum samples of healthy pregnant women during second trimester. Their results demonstrated that 98% of women were EBV-seropositive with reactivation rate of 35% without adverse pregnancy outcomes (28). In a prospective cohort study on VZV over a 13-year period, the fetal infection rate was 0.8% but the prevalence of congenital varicella syndrome was 0.39% (29). Different rates of HSV1/2 infections have been reported in diverse studies. It was shown that intrauterine HSV1/2 infections caused growth restriction of the fetus, preterm delivery, and an increased risk of spontaneous abortions (15). Formerly, a study in Iran performing PCR on the placental tissue and curettage specimens of 35 full-term delivery cases and 35 spontaneous abortion casesshowed that HSV DNA was detected only in 2.8% of spontaneous abortions. They could not detect any significant differences between the case and control groups (30).

## 5. Conclusion

In conclusion, here, no correlation was detected between the HHVs placental infections and increased risk of spontaneous abortions. The divergent study results that have been reported in regards to HHVs placental infections and increased risk of spontaneous abortions could be due to the different types of specimens, populations, and differences in specificity and/or sensitivity of assays. In order to find the actual role of HHVs infection in this regard, further investigations should be performed with larger sample size and on populations in different countries.

##  Conflict of Interest

The authors declare no conflict of interests regarding the publication of this paper.
